# COVID-19 Vaccine Booster-Induced Dermatographism

**DOI:** 10.7759/cureus.26566

**Published:** 2022-07-05

**Authors:** Mohammad A Amjad, Zamara Hamid, Pius Ochieng, Si Li

**Affiliations:** 1 Internal Medicine, The Wright Center for Graduate Medical Education, Scranton, USA; 2 Medicine, Shifa International Hospital, Islamabad, PAK; 3 Pulmonary and Critical Care Medicine, Geisinger Community Medical Center, Scranton, USA

**Keywords:** morbilliform drug eruption, chronic spontaneous urticaria, cutaneous reactions, covid 19 vaccine, dermatographism

## Abstract

The urgent requirement for a preventative vaccination became more pressing due to the severe repercussions that the SARS-CoV-2 (COVID-19) virus had on society and the economy. The deployment of the COVID-19 vaccination program had to be expedited. As with all vaccinations, adverse events have been recorded with the COVID-19 vaccine. Some patients may experience cutaneous reactions such as rashes, itching, hives, and swelling after receiving the COVID-19 vaccine, but it is unclear how common these events are or how frequently they recur. This article discusses an unusual case of a young man who got chronic severe dermatographism after receiving a booster shot of the Moderna vaccine (Moderna, Inc., Cambridge, Massachusetts).

## Introduction

Dermatographism refers to urticarial eruptions generated by pressure or mechanical tension on the skin. Dermatographism is the most common type of inducible urticaria, affecting between 2% to 5% of the population [1]. Although the specific etiology of dermatographism is unknown, the most frequently accepted explanation is that the unstable mast cells produce histamine, bradykinin, leukotrienes, and peptides. There are several forms of dermatographism, notably follicular dermatographism (small punctate wheals), red dermatographism (isolated urticarial papules), and familial cholinergic dermatographism (extensive erythematous line with punctate wheels) [1]. Cutaneous symptoms, including rashes, itching, and swelling, have been recorded [2,3]. This is a case of a patient who developed persistent severe dermatographism after receiving a booster shot of the Moderna vaccine (Moderna, Inc., Cambridge, Massachusetts).

## Case presentation

**Figure 1:**
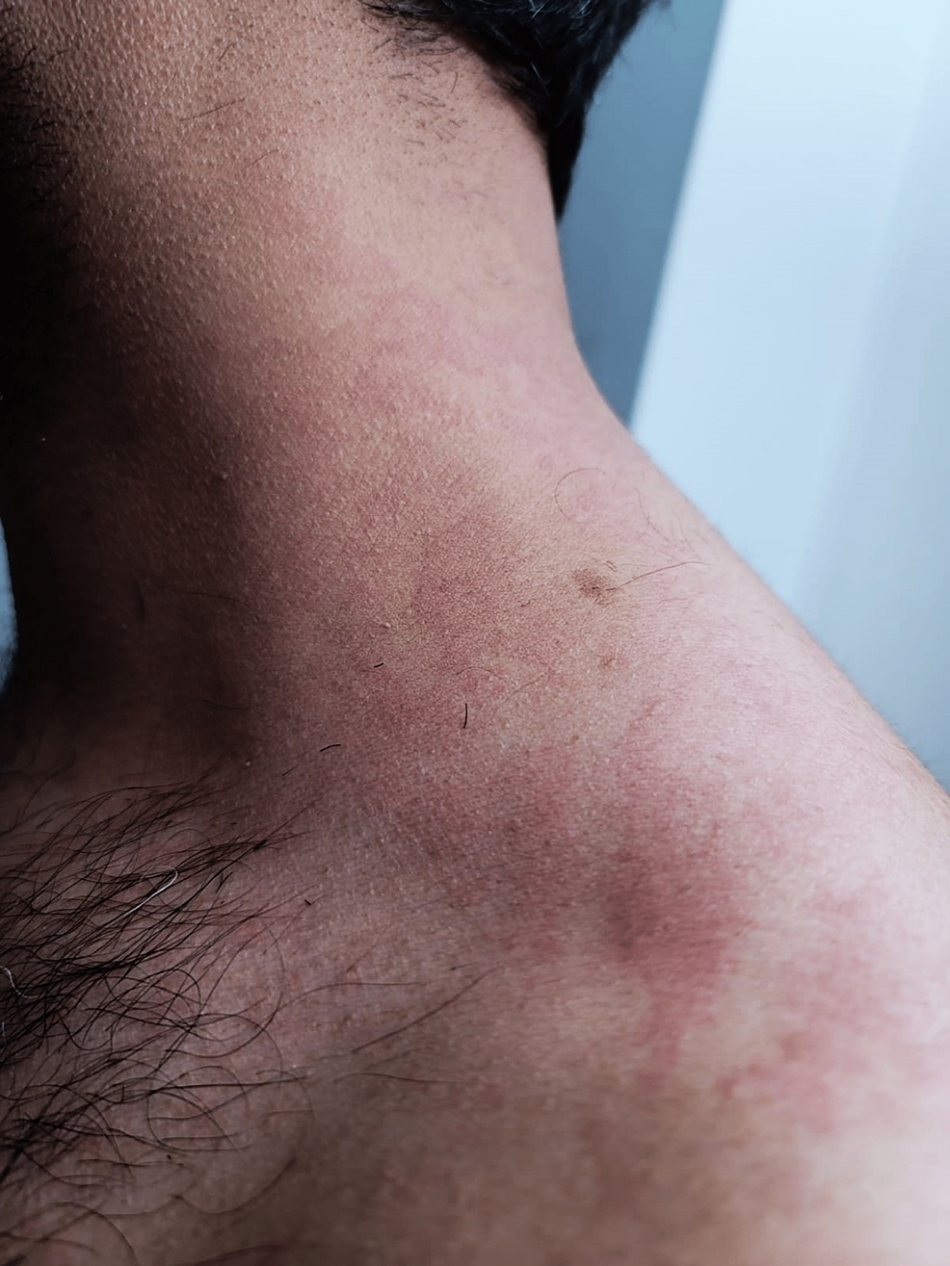
Raised, red wheals on the skin The figure was generated completely for this publication and gained agreement from the patient to post it

A healthy 31-year-old nonsmoker with no known allergies had itching and raised wheals in his bilateral forearms, thighs, and ankles for one week. The itchy wheals go away in a couple of minutes without scarring, but scratching and hot baths worsen them. He denied palmar, plantar, face, and genital lesions and any chemical or solvent exposure. Also, no systemic symptoms were linked with these episodes, such as lip/tongue swelling, dyspnea, and dysphagia. He had a Moderna coronavirus disease 2019 booster vaccine two weeks before the presentation. Following the initial two Moderna vaccinations, he had not encountered any adverse reactions. He denied having recently traveled, been exposed to COVID-19 at work, or used new personal care items. He had tried an over-the-counter course of loratadine, but it had failed to provide relief. On physical examination, there was a severe maculopapular rash on the upper limbs (Figure 1) and striae on the abdomen. He exhibited no swelling on the tongue, lips, or throat, and his lungs were both clean on auscultation.

**Figure 2:**
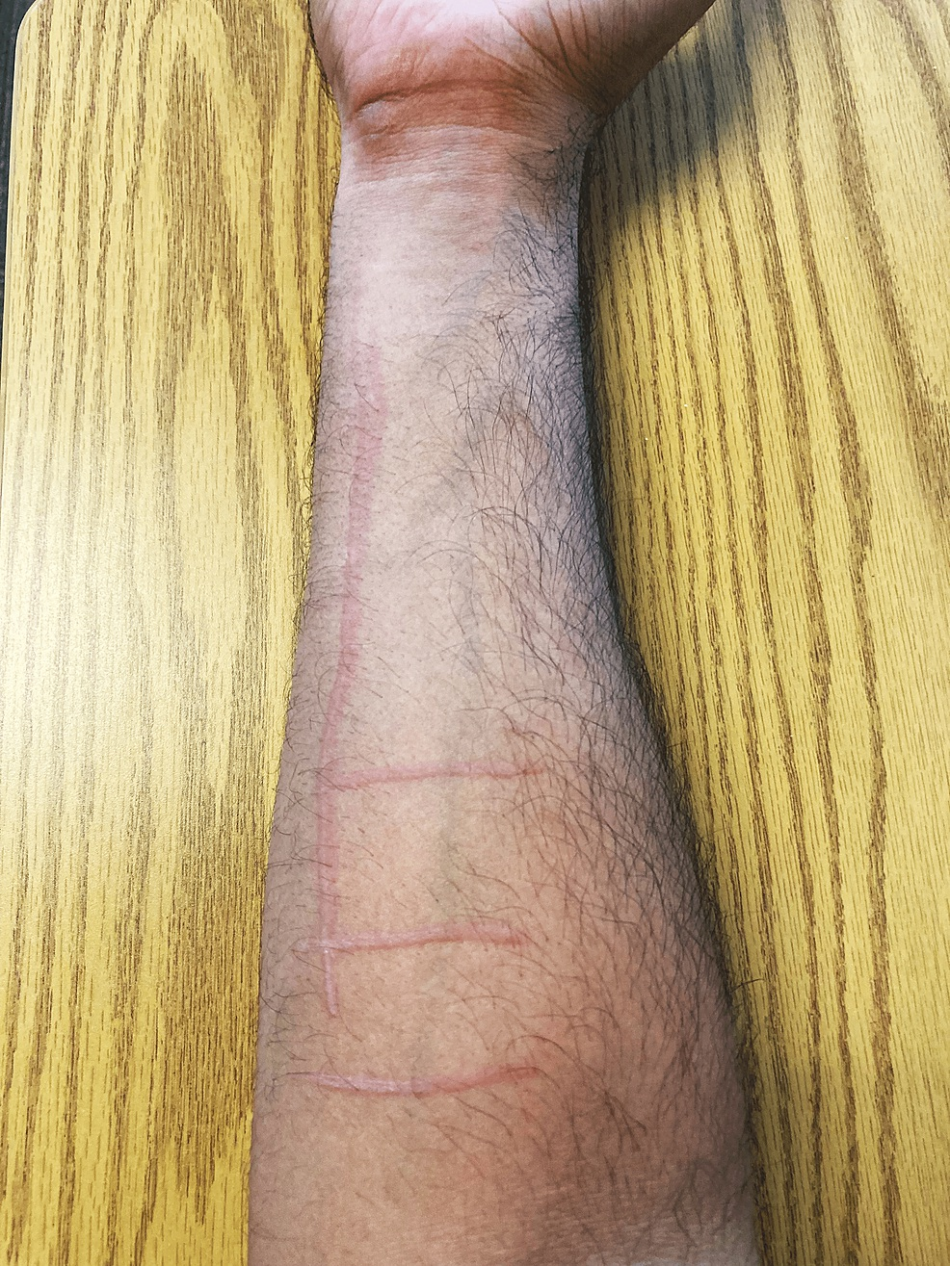
A tongue blade was firmly drawn across the right forearm's distal flexor surface, which produced a wheal and flare reaction in less than one minute: a positive Darier sign The figure was generated completely for this publication and gained agreement from the patient to post it

In less than one minute, wheal and flare lesions appeared on his right forearm after a gentle tongue depressor stimulation (Video 1, Figure 2).

**Video 1:** Positive Darier sign

The results (Table 1) of standard laboratory tests (complete blood count, urinalysis, and comprehensive metabolic profile), as well as IgE level and allergens testing (Table 2), were unremarkable. Also, he was COVID-19 negative. After four weeks of oral prednisone 40mg daily with tapper and an oral cetirizine 10 mg once daily, his signs and symptoms were adequately managed. He continues to take an oral antihistamine, with follow-ups every six months. On the other hand, spontaneous urticaria continues to occur as soon as the patient stops taking the antihistamine dose. 

**Table 1:** Laboratory work-up

Test name	Patient values	Reference range	Units
White blood cell count	8.0	4.0-10.80	K/uL
Red blood cell count	5.24	4.00-5.25	M/uL
Hemoglobin	15.2	14.0-16.8	g/dL
Hematocrit	44.2	40.0-48.4	%
Red cell distribution width (RDW)	13.1	11.5-15.5	%
Mean corpuscular volume (MCV)	84	82.0-99.5	fL
Mean corpuscular hemoglobin	29.0	27.0-34.0	Pg
Mean corpuscular hemoglobin concentration	34.4	32.0-36.0	g/dL
Platelet count	107	140-400	K/uL
Monocytes	3.9	1.0-11	%
Neutrophils	61.0	40.0-75.0	%
Lymphocytes	30.0	18.0-42.0	%
Eosinophils	3.0	0.0-6.0	%
Absolute basophils	0.0	0.0-0.2	K/uL
Absolute eosinophils	0.2	0.0-0.7	K/uL
Absolute lymphocytes	3.25	1.0-4.8	K/uL
Absolute monocytes	0.7	0.3-1.0	K/uL
Absolute neutrophils	4.7	1.8-7.8	K/uL
C-reactive protein	1.5	<3.0	mg/dl
Erythrocyte sedimentation rate	20	0-35	mm/h
Urea	24	15-39	mg/dl
Creatinine	1.10	0.57-1.11	mg/dl
Sodium	142	135-146	mEq/L
Potassium	4.9	3.5-5.1	mEq/L
Phosphorus	3.5	2.5-4.9	mg/dl
Total bilirubin	0.29	0.2-1.0	mg/dl
Lactate dehydrogenase	215	84-246	UI/L
Alanine aminotransferase	42	30-65	UI/L
Aspartate aminotransferase	23	15-37	UI/L
Albumin	3.0	3.4-5.0	g/dl
Thyroxine (T4)	1.21	0.76-1.46	ng/dL
Thyroid-stimulating hormone	0.650	0.358-3.740	UI/mL
Folic acid	15	3.1-17.5	ng/dL

**Table 2:** Allergens profile Allergen-specific immunoglobulin E (IgE) concentration interpretation (kU/L) < 0.10 negative; 0.10-0.35 borderline; 0.35-0.69 low positive; 0.70-3.4 moderate positive; 3.5-17.4 high positive; 17.5-49.9 very high positive

Test Name	Result	Reference range	Unit
Immunoglobulin E, total	73	< 100	IU/mL
Allergen (F20) IgE almond	< 0.10	< 0.35	kU/L
Allergen (F18) IgE brazil nut	< 0.10	< 0.35	kU/L
Allergen (F202) IgE cashew nut	< 0.10	< 0.35	kU/L
Allergen (F17) IgE hazelnut/filbert	< 0.10	< 0.35	kU/L
Allergen (F13) IgE peanut	< 0.10	< 0.35	kU/L
Allergen (F201) IgE pecan nut	< 0.10	< 0.35	kU/L
Allergen (F203) IgE pistachio	< 0.10	< 0.35	kU/L
Allergen (F256) IgE walnut (black, english)	< 0.10	< 0.35	kU/L

## Discussion

Dermatographism is an urticarial eruption characterized by a linear wheal in the form of the externally applied force. It's also called dermographism urticaria and urticaria factitia. Writing on the skin is a literal translation of this phrase. It is the most prevalent form of inducible physical urticaria, affecting 2% to 5% of the population [1]. It is commonly noticed in young adults, with a peak occurrence in the second and third decades. People who have had stressful life events (pregnancy, menopause), psychological co-morbidities or infections (influenza A), or certain medications or vaccinations use are more likely to have dermatographism [4,5].

Food and Drug Administration (FDA) issued emergency use authorization for both the Moderna and Pfizer-BioNTech (Pfizer Inc., New York City, New York; BioNTech SE, Germany) vaccines in response to the ongoing coronavirus disease-2019 (COVID-19) pandemic [6]. According to the Centers for Disease Control (CDC) prescribing guidelines for vaccinations, a severe allergic reaction to a prior dose of the vaccine or any component of the vaccine is a contraindication to immunization. These vaccines have a high efficacy in preventing symptomatic COVID-19 infection, and they are associated with a low risk of adverse events, such as local injection site reactions and rare occurrences of myocarditis and pericarditis [2,3].

A prospective cohort of over 50,000 health care professionals reported cutaneous responses after receiving the first dose of a messenger RNA (mRNA) COVID-19 vaccination. There were no recurrence cutaneous reactions among the more than 600 personnel who experienced first-dose cutaneous reactions. Another 2.3% developed skin reactions after the second immunization dose [7]. However, little is known about the skin responses related to booster immunizations. Although rare in certain studies, delayed skin reactions are the most common dermatologic side effect, especially after the Moderna COVID-19 (mRNA-1273) booster vaccine, as also evident in our case [8-11]. He did not exhibit any adverse reaction to the first or second dose of the Moderna COVID-19 (mRNA-1273) vaccine but developed delayed urticarial eruptions after receiving a booster shot.

The precise etiology of dermatographism is unknown; however, the most frequently accepted hypothesis is that it is caused by the release of histamine from mast cells in reaction to an allergen, together with bradykinin leukotrienes and peptides [12]. Polyethylene glycol (PEG) has been suggested as an allergen causing these allergic reactions in the COVID-19 mRNA vaccination [13,14]. As a result, the "Lewis triple reaction" may develop, including early capillary dilatation, leading to a superficial erythematous phase, followed by erythema and fluid transudation to form the linear wheal. The complete response could take up to five minutes [12].

It can be incredibly challenging to manage symptomatic dermatographism. Preventing and minimizing triggering variables such as physical stimulation, as well as lowering stresses, are critical in the treatment of dermatographism [1]. The majority of individuals are asymptomatic; therefore, treatment should be restricted to those with symptoms. H1 antihistamines such as cetirizine or loratadine are effective treatments. If H1 blockers are insufficient to control the pruritus, other therapeutic options include H2 antihistamines, leukotriene antagonists, cyclosporine, and oral steroids [5].

COVID19-induced cutaneous reactions have been observed to be self-limiting [3,7,8]. Nevertheless, according to a study by Thomas et al., the patient had chronic spontaneous urticaria following vaccination and, like the patient in our case report, continues to experience daily cutaneous symptoms despite H1 antihistamine administration [11].

## Conclusions

This case study focuses on a young man who received a booster dose of the Moderna mRNA-1273 COVID-19 vaccine and afterward experienced dermatographic urticaria. Long-term cutaneous responses have not been linked to anaphylaxis or other symptoms of sudden allergic reactions, so they shouldn't stop people from getting immunized. It is necessary to conduct additional research to comprehend the mechanism behind chronic dermatographic urticaria after receiving a booster shot of COVID-19 and the medical treatment for it.
